# Dentulous versus edentulous mandibles: CBCT-based morphometric assessment of mandibular canal and alveolar bone

**DOI:** 10.4317/jced.59033

**Published:** 2022-12-01

**Authors:** Thekra-Ali Saeed, Ameenah-Saad Alansy, Zainab-Abdulkader Abdu, Osama Almaqtari, Zhanhai Yu

**Affiliations:** 1 School of Stomatology, Lanzhou University, Lanzhou 730000, Gansu Province, China; 2 Faculty of Dentistry, University of Science & Technology, Sana’a, Yemen

## Abstract

**Background:**

Precise preoperative knowledge of the mandibular canal (MC) variations and alveolar bone dimensions are vital elements for a successful dental implant. Thus, this study aimed to describe the three-dimensional morphology of the MC and alveolar bone dimensions, followed by a comparison of the variables in edentulous mandibular posterior sides with contralateral dentulous sides among adult Chinese individuals. Such variations were also studied in relation to gender and age.

**Material and Methods:**

Cone-beam computed tomography (CBCT) was used for the morphometric analysis of the MC and alveolar bone dimensions in this cross-sectional study that analyzed retrospectively gathered data. Records of 112 individuals (56 males and 56 females) who had one edentulous mandibular posterior side and one dentulous side were included in the analysis. The MC position, length (MCL), and diameter (MCD) along with superior bone height (SBH) and bone width (BW) of the alveolar bone ridge were assessed.

**Results:**

The MCD was lower at first (*p* = 0.016) and second (*p* = 0.079) molars on the edentulous sides. The SBH, BW1mm, and BW3mm were significantly lower on the edentulous than dentulous sides (*p*< 0.05). However, there were no significant differences in the MC position or MCL and the incidence of bifid MC for dentulous and edentulous sides. Gender was a significant parameter for MCL, SBH, and BW, while no significant differences were observed in all variables on both sides in relation to age.

**Conclusions:**

The position of the MC remains relatively constant regardless of losing teeth or increasing age. However, the MC position and MCL show differences in relation to gender. Alveolar bone dimensions are highly affected by dentate status followed by gender. Therefore, such variations should be considered by surgeons for successful surgical procedures in the posterior mandible.

** Key words:**Mandibular canal, Bifid mandibular canal, edentulous mandible, alveolar bone dimension.

## Introduction

The mandible forms the lower part of the face and is composed of the body and two rami (1). The posterior region of the mandible is highly vascularized due to the presence of the inferior alveolar bundles and the submandibular fossa, making it vulnerable during implant installation, third molar surgery, mandible osteotomy, or endodontic treatments (2,3). Successful dental implantation in posterior mandible mostly depends on two parameters for the long-term stability of the implant: protection of the inferior alveolar nerve (IAN) and sufficient alveolar bone volume (4,5). Thus, accurate knowledge of the posterior region of the mandible is ineviTable to protect the IAN from any iatrogenic damage during invasive surgical procedures (3,6,7). Morphological variations in the mandibular canal (MC) are highly influenced by the differences in status, age, society, degree of mandibular atrophy, and the technique of assessment (1,8,9). On the other hand, the volume and rate of alveolar bone resorption depend on several factors such as mechanical, nutritional, environmental, age, gender, and race. Systemic factors, including hormonal disturbance, metabolic bone disorders, and postmenopausal hormonal imbalance, contribute to residual ridge resorption and can alter mastication efficiency and mandibular morphology (10,11). Dental-associated factors, including periodontitis, periapical pathosis, dental fracture, and traumatic extraction, could lead to alveolar bone damage (5,12,13).

The position of anatomical structures and alveolar bone dimensions may change with tooth loss (13,14). Differences in bone loss patterns have been reflected in the availability of bones for implant therapy in edentulous patients. Accordingly, implant placement requires knowledge of MC anatomy and proper assessment of bone quality and quantity to help choose the suiTable size and right position of dental implant and to reduce the risk of IAN damage (4,7). Therefore, this study aimed to describe the position and course of the MC as well as alveolar bone dimensions on edentulous mandibular posterior sides and contralateral dentulous sides using cone-beam computed tomography (CBCT) images. The variations in MC and alveolar bone dimensions were also investigated by gender and age.

## Material and Methods

This is a cross-sectional study that analyzed retrospectively gathered data was conducted at the Hospital of Stomatology, Lanzhou University, China after obtaining approval from the Ethics Committee of the hospital (No. LZUKQ-2019-049). CBCT scans of adult Chinese patients, who required the images for diagnosis/dental treatment, were retrieved from CBCT records between October 2019 and November 2020. However, 112 CBCT scans were included in the study based on the presence of one edentulous mandibular posterior side and one dentulous side if the patients were aged between 38 and 65 years and had lost all molar teeth on one mandibular side but had all molar teeth in the contralateral mandibular side, and the scans were normal at the location of measurements. However, the CBCT scans were excluded from analysis if the patient had pathological lesions as indicated by a radiolucent or a radiopaque zone, had a dental implant on the edentulous side, or had recent tooth extraction (internal borders of alveolar sockets identifiable in the CBCT), or the images showed unclear anatomical structures.

The sample size of the present study was calculated according to Pramstraller *et al*. (5) at an alpha value of 0.05 and a statistical power of 95%. The mean value of the bone height at the first molar was set at 16.08±2.96 mm and 13.41±2.82 mm on the dentulous and edentulous sides, respectively. Based on this calculation, the sample size for our study was suggested to be 32 patients in each group. However, 56 CBCT images were included in each group to increase reliability of the measurements.

The CBCT images were divided into two groups according the dentate status (112 dentulous sides and 112 edentulous sides) and gender (56 males and 56 females) of individuals, while they were divided into three groups according to the age of individuals (38-48 years, 49-59 years and 60-65 years).

As per records, CBCT examination was performed using the i-CAT Imaging System (Imaging Sciences International Inc., Hatfield, PA, USA), and all the patients were scanned with a standard protocol: field of view (FOV) of 16.0 × 13.0 cm), the exposure parameter setting (tube voltage =120 kVp, a filament current = 18.54 mAs, a total scan time = 8.9 seconds), and image acquisition at 0.3 mm voxel size. The CBCT images were then analyzed using the Invivo software, Version 6 (Anatomage, San Jose, CA, USA). Initially, the position of teeth on the edentulous side was identified. In the pano section of the arch section view, the distances between the long axis for each tooth of the dentulous side and the center (midline) of the CBCT were measured. The same distances in the contralateral edentulous side were then determined to identify the positions of missing teeth because the distance from the center of the tooth to the middle of CBCT in the dentulous and edentulous sides was symmetric (5).

**Figure 1:**
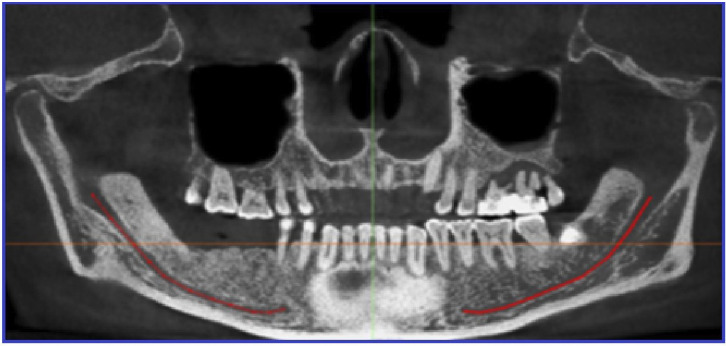
Length of the mandibular canal (MCL) in panoramic view; the mandibular foramen and the mental foramen are marked, curved distance between the two foramina is measured in mm where the red line crosses between the upper and lower cortication of the canal.

The following variables of CBCT scans were assessed according to previous studies (7,8,10,15):

Length of the mandibular canal (MCL): This was measured in the panoramic view along the line passing between upper and lower cortication of the MC from the mandibular foramen to the mental foramen (Fig. 1).

**Figure 2:**
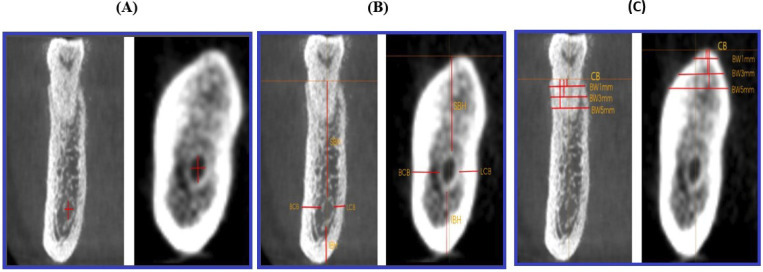
Measurements of maximum vertical and horizontal diameters of the mandibular canal (MC) in dentate and edentulous sides (A); Measurements of superior bone height (SBH), inferior bone height (IBH), buccal cortical bone (BCB) and lingual cortical bone (LCB) on the dentulous and edentulous sides (B); and bone width measurements at three heights (1mm, 3mm, and 5mm), the reference lines at the three points (the vertical lines) below the coronal point of alveolar bone (CB) and the radiographic measurements (the horizontal lines) from buccal to lingual margins on the dentulous and edentulous sides (C).

Diameter of the mandibular canal (MCD): The maximum vertical and horizontal diameters were measured in the coronal plane as the distances between the inner surfaces of the MC border in the tooth roots of the dentulous side and the tooth site of the edentulous side (Fig. 2A).

Location of MC: The distances of the buccal cortical bone (BCB) and lingual cortical bone (LCB) were measured on dentulous and edentulous sides (Fig. 2B).

Superior bone height (SBH): The distance from the outermost coronal margin of the MC to the most coronal point (CB) of the alveolar crest (in dentulous sides) or the alveolar ridge (in edentulous sides) was measured (Fig. 2B).

Inferior bone height (IBH): The distance from the most apical margin of the MC to the inferior border of the mandible was measured (Fig. 2B).

Bone width (BW): This was measured on coronal slices from buccal to lingual border at three points (1 mm, 3 mm, and 5 mm) below the CB on dentulous/edentulous sides and recorded as BW1mm, BW3mm, and BW5mm, respectively (Fig. 2C). The BW was not calculated in the edentulous slices when the reference lines were traversed or below the MC, while it was not measured in the dentulous slices if the vestibular and/or lingual bone was not found. All measurements were performed with a digital ruler at 0.01 millimeters (mm) increments.

**Figure 3:**
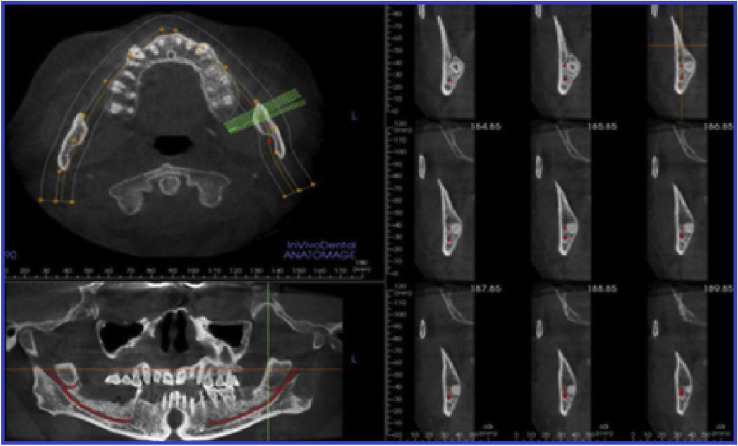
Assessment of bifid mandibular canal (BMC) in axial, cross-sectional, and panoramic views.

Bifid mandibular canal (BMC): Coronal, axial, cross-sectional and panoramic views were monitored to assess the BMC, which was subsequently categorized based on the source site and course according to Naitoh’s classification (16) (Fig. 3).

For the reliability of measurements, all variables were measured by two previously trained examiners, and the inter-rater agreement was calculated. Then, the interclass correlation coefficient (ICC) was calculated using a repeated measurement after one month of initial analysis. The reliability analysis exhibited an excellent inter-rater agreement (0.91) and an ICC of 0.94.

Data were analyzed using the IBM SPSS Statistics for Windows, Version 25.0 (IBM corp., Armonk, NY, USA). The mean value and standard deviation (SD) of each measured variable were calculated, and the normality of data distribution was tested using the Shapiro-Wilk and Kolmogorov-Smirnov tests. The mean values of the measured variables were compared according to the dentate status and gender using the independent samples Student’s t-test and the paired samples Student’s t-test, as appropriate. However, analysis of variance (ANOVA) was used to compare the means of the measured variables according to the age groups. Statistical significance was considered at *p-value*s <0.05.

## Results

The mean value of MCL was 62.83±4.43 mm. The lowest mean value of the MCD was observed in the first molar region that gradually increased until the third molar region on both dentulous and edentulous sides. The vertical diameter of the MC was higher than the horizontal diameter, being 2.93 mm and 2.19 mm, respectively. Based on the thickness of cortical bone, the position of MC was close to the buccal cortical in the first molar, while it was close to the lingual cortical bone in the second and third molars. BMC was observed in 11 cases (9.8%), while the bilateral bifid canal was observed in one case. According to Naitoh’s classification, the most common type of BMC was the retromolar canals (45.5%), followed by the forward (36.4%) and dental (18.1%) canals.

The maximum mean values of SBH were observed in the first molar region of the dentulous and edentulous sides (16.74±2.61 mm and 14.60±3.08 mm, respectively), which decreased posteriorly from the first molar. On the other hand, the maximum mean value of IBH (8.81±1.85 mm) was observed in the third molar region. The BW at the three positions (1mm, 3 mm, and 5 mm) significantly increased from the first to third molars. Overall, BW significantly increased from the coronal to apical (BW1mm < BW5mm) in all positions, the mean value increased from 10.68 mm to 13.56 mm on the dentulous side and from 6.97 mm to 13.19 mm on the edentulous side.

There was no statistically significant difference between the mean values of the MCL on both dentulous and edentulous sides (62.85±4.08 mm vs. 62.81±4.75 mm, respectively; *p* = 0.947). Compared to the edentulous side, the MCD of the dentulous side was significantly higher (*p*=0.016) at the first molar, but non-significantly higher (*p* = 0.079) at the second molar. The BCB and LCB thickness showed no significant difference between edentulous and contralateral dentulous sites at all positions ( Table 1). SBH, BW1mm, and BW3mm were significantly lower in the edentulous compared to dentulous sides at all positions. Conversely, IBH did not show any significant difference between the edentulous and dentulous sides at all positions ( Table 2).

**Table 1:**
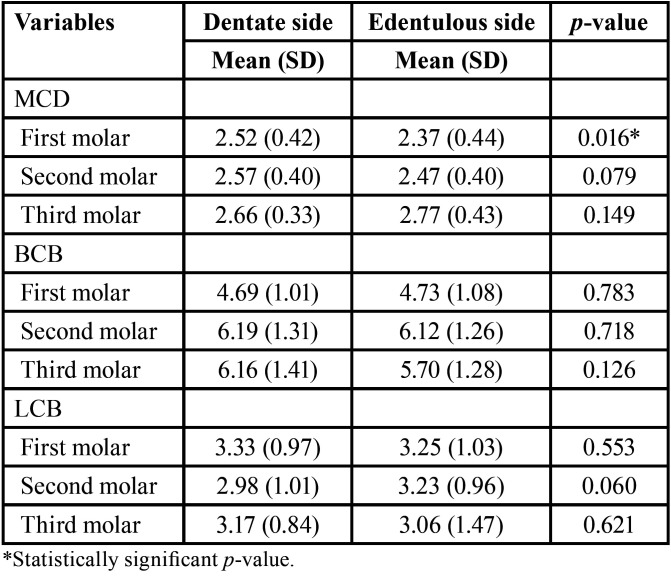
Diameter of the mandibular canal (MCD), buccal cortical bone (BCB) and lingual cortical bone (LCB) measurements (in mm) in dentulous and edentulous sides.

**Table 2:**
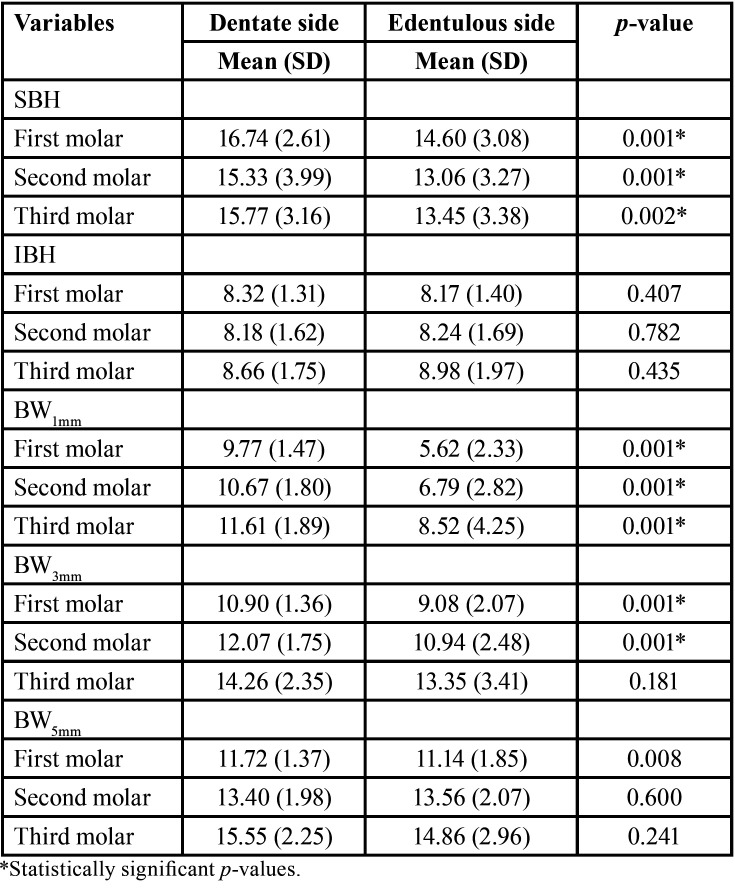
Superior bone height (SBH), inferior bone height (IBH) and bone width (BW1mm, BW3mm, and BW5mm) measurements (in mm) at dentulous and edentulous sides.

The MCL was significantly higher in males than females (64.55±4.089 mm vs. 61.11 ±4.079 mm, respectively), The MCD showed a statistically significant difference at the second (*p* <0.001) and third (*p* = 0.050) molars. The BCB was significantly thicker in males than females at the first molar (*p*=0.046), while the LCB was significantly thicker in females at the second (*p* <0.001) and third (*p* = 0.001) molars. Of 112 cases, BMC was observed in six males (10.7%) and five females (8.9%) ( Table 3). Females showed lower mean values of SBH, BW1mm, and BW3mm on the dentulous and edentulous sides than males. However, IBH was not significantly different between males and females, except at the first molar (*p* = 0.020) ( Table 4). There were no statistically significant differences in the mean values of all variables on the dentulous and edentulous sides in relation to the age of patients.

**Table 3:**
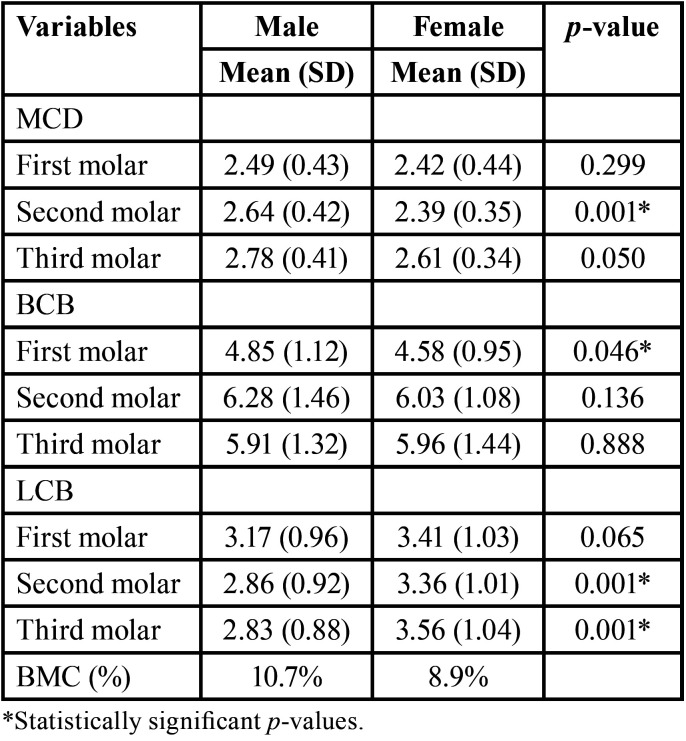
Diameter of the mandibular canal (MCD), buccal cortical bone (BCB) and lingual cortical bone (LCB) measurements (in mm) and the prevalence of bifid mandibular canal (BMC) in relation to gender.

**Table 4:**
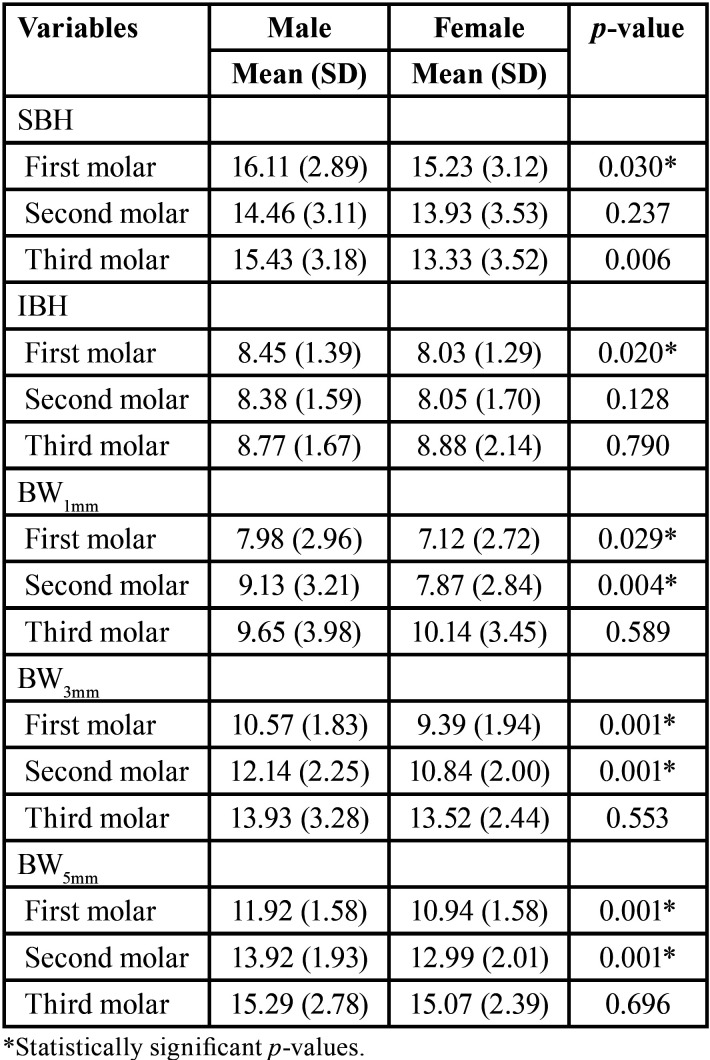
Superior bone height (SBH), inferior bone height (IBH) and bone width (BW1mm, BW3mm, and BW5mm) measurements (in mm) in relation to gender.

## Discussion

The assessment of the MC morphology and alveolar bone dimensions in the mandibular posterior region is critical before dental implant placement to prevent any injury in the IAN and obtain long-term stability of the dental implant. Therefore, the present study assessed these two parameters among adult Chinese patients subjected to CBCT scanning. The significantly higher MCL in males than females in the present study is consistent with the difference of 3.04 mm reported for Turkish patients with no statistically significant difference by gender (17). The present study also revealed a lower MCD on the edentulous compared to dentulous sides. This finding is in line with that the MCD becomes smaller after tooth loss (5,7). The present study showed an oval-to-round shape of the MC, where the MCD increased from the first molar posteriorly on both sides. This finding is consistent with the literature (8,18). The significant association of gender with MCD in the present study agrees with that reported among the Jordanian population (19). It is noteworthy that gender plays an important role in the resorption of the MC wall (11,14).

The buccolingual location of MC is an important parameter that should be assessed before any surgical procedures (8). In the present study, the BCB was thickest at the distal root of the second molar (6.33±1.33 mm) and thinnest at the mesial root of the first molar (4.38±1.05 mm). Conversely, the LCB was thinnest at the distal root of the second molar (2.96±1.04 mm), and thickest at the mesial root of the first molar (3.41±1.07 mm). Sirbu *et al*. (20) found that the distance between the MC and the buccal margin of the mandible gradually increased from the second premolar to the second molar, while the distance between the MC and lingual margin decreased from the second premolar to the second molar. The appropriate position to perform the vertical anterior osteotomy is between the first and second molars because of the thicker buccal bone in this region (19,21). In the present study, gender was significantly associated with variations in the BCB and LCB, showing that bone size and thickness may be affected by genetic, systemic and environmental factors (15,19). The lack of age difference in the MC in the present study is consistent with previous studies (17,21,22). In contrast, age was found to be a significant parameter for the location of MC and bone thickness in the region of mandibular sagittal split osteotomy among the Jordanian cases (19). The course of MC passes from the buccal plate at the first molar lingually towards the second and third molar. Arias *et al*. (3) also observed that the courses of the MC pass from the vestibular cortical anteriorly towards the lingual cortical in the posterior mandibular body. Meanwhile, Nimigean *et al*. (7) observed the same course of MC when analyzing CBCTs and dry mandibles of edentulous subjects. The present study showed that the dentate status did not affect the BCB and LCB thickness at all positions. Previous studies concluded that the distance between MC and the buccal and lingual borders does not change with tooth loss and at any stage of the atrophic process of the mandible (7,23). The position of the MC remains relatively constant regardless of losing teeth or increasing age.

In the present study, the prevalence of BMC (9.8%) almost matches with that reported among Korean and Chinese individuals, being 10.2% and 13.2% respectively (24,25). In contrast, Naitoh *et al*. (16) reported a higher prevalence of 64.85 for BMC using CBCT. This could be due to differences in regional or racial factors, CBCT machines, radiographic software, and radial assessment techniques. Overall, the incidence rate of BMC is similar for populations existing in related areas. Moreover, the incidence of BMC has not been found to be significantly different in relation to gender or dentate status (24-26). According to Naitoh’s classification, the most common types of BMC among Chinese people were the retromolar and foreword canal (25), while the least common type was the buccolingual canal (2.86%) (27).

The gradual decrease in SBH and increase in BW from the first molar posteriorly on the edentulous sides in the present study aligns with that reported in the literature (4,10). Several studies showed significant differences in bone height and width between dentulous and edentulous subjects (4,5,10,12). IBH did not show any significant difference between the edentulous and dentulous sides in the present study which is in line with that reported among Italian patients (5). The lower values of SBH and BW in females compared to males are consistent with those reported previously for dentate (3,28) and edentate subjects (4,10,29). Promma *et al*. (21) reported thicker inferior cortical bone in females than males in the premolar region. This is mainly associated with hormonal effects such as postmenopausal estrogen depletion or hyperthyroidism, which affect the calcium metabolism (10,29). In the current study, no significant age-related differences in the SBH, IBH, and BW on the dentulous and edentulous sides. Age was not found to significantly influence BH and BW of the posterior edentulous regions in previous studies (4,10). The lack of significant variations in age groups could be attributable to the parafunctional conditions in which muscular hyperactivity protects bone from excessive resorption (13).

The position of MC observed in the present study and other populations of India (8), Chile (1), Romania (7), and America (22) was almost similar. The alveolar bone dimensions in the present study are in line with those reported from Switzerland (4), Italy (10), Turkey (13) and Thailand (21), where the alveolar bone was found to be highly affected by the dentate status and gender. Accordingly, the geographic location does not seem to have a significant effect on the MC and alveolar bone measurements; however, the differences in measurements might be applicable to other ethnicities.

This study is limited by the retrospective nature of its design. Therefore, the medical and dental history of the patients, denture wearing, time of tooth extraction, and intake of medications were not available in the retrieved records, which could have influenced the dentate status and/or gender-related measurements. Nevertheless, the findings of the present study provide insights into the CBCT-based morphometric differences between the MC and alveolar bone dimensions in the mandibles in relation to dentate status, gender and age.

## Conclusions

The position of the MC remains relatively constant regardless of losing teeth or increasing age, while it is slightly affected by gender. On the other hand, alveolar bone height and width are highly affected by dentate status followed by gender. Hence, such variations should be considered by surgeons for successful surgical procedures in the posterior mandible.
